# Efficacy of the open anterior prosthetic repair in the surgical management of recurrent inguinal hernia in old patients

**DOI:** 10.1186/1471-2482-13-S1-A17

**Published:** 2013-09-16

**Authors:** Giovanna Brancato, Maurizio Ristagno, Angelita Giglio, Antonio Biondi, Marcello Donati

**Affiliations:** 1 Department of Surgical Sciences, Organ Transplants and New Technologies.General Surgery and Week Hospital Unit. University Hospital of Catania. Via S.Sofia 78. 95123, Catania. Italy; 2 Department of Surgery. General and Oncologic Surgery Unit. Vittorio-Emanuele University Hospital of Catania. Via Plebiscito 628. 95122, Catania, Italy

## Background

To date no standard treatment for recurrent ingunal hernias exists. Open or laparoscopic approaches were proposed with apposition of meshes with reknown advantages and limits[1]. Long term recurrence-rate, postoperative pain, fistulization or prosthetic infection risks remain open questions in this surgery. Aim of this work is to show results of our experience with the open anterior approach in local anesthesia[2] for recurrent inguinal hernia repair in old patients (Age>75 yrs.).

## Materials and methods

Between January1994 and December 2011, on a total of 3120 patients referred to us for inguinal hernias, 218 patients (6.9%) were submitted to inguinal hernioplasty for hernia recurrence. Twenty-nine (13.3%) of them were <75 years old, with a middle age of 80.59 (range 75-89). The time for recurrence appearance was ranging from 3 to 191.5 months. Ten (34.4%) were recurrence after mesh implantation, while 19 (65.6%) were after traditional hernioplasty for direct suture. They were all affected by one or more comorbidities: Sixty patients (55.1%) by cardiovascular disease, 8 by respiratory disease (27.5%) , 3 by neuropathy (10.3%), 6 by metabolic one (20.7%). They underwent an inguinal hernioplasty by open anterior approach with prosthetic material (polypropylene) apposition in 26 patients (89.6) under local anesthesia, in 2 patients (6.9%) under spinal anesthesia and in 1 more patient (3.5%) under general anesthesia.

## Results

We repaired in 12 cases (41.3%) by apposition of a cilindric Lichtenstein’s plug[3], in 6 cases (20.7%) with Trabucco’s T1 plug, in 9 cases (31.1%) by performing a mesh and plug repair and in 2 cases (6.9%) with a “mesh only” repair. Only polypropylene mesh or plug were used[4]. No drainage was used. We registered no intraoperative and postoperative complications. Twenty-four patients (82.7%) were discharged within 24 hours. In a long-term telephonic, clinical and ecographic follow-up (middle value 107 months; range 16-220 months) we found 2 recurrences (~7%). Those patients were again repaired through an open anterior approach. No mesh infections or fistulas, so like chronic pain cases were registered in our follow-up. Never was necessary to remove a plug or a mesh.

## Conclusions

The recurrent inguinal hernia repair in old patients (>75 yrs), due to general aging of population and number of inguinal hernioplasties all over the world, is becoming a clinical entity of moderate impact on surgeon’s daily activity (about 13% of inguinal hernioplasties for recurrences in our experience). Recurrent hernia in old patients is often present togheter with important comorbidities, expecially cardio-respiratory ones. An open anterior approach in local anesthesia shows advantages of really “mini-invasive” method, with reduction of surgical risks[5], in front of laparoscopic techniques in which general anesthesia is usually required. Other advantages are prompt reahabilitation, feasability in most of case in day surgery[6] setting and good long-term results about recurrence if compared with till today published literature’s data. Additionally as our experience shows, in case of re-recurrence, patients are more prone to accept a new operation due to reduction of trauma of the first intervention.

In conclusion recurrent inguinal hernia repair requires an eclectic surgical approach, in old patients anterior approach with polypropylene mesh, or plug, or both apposition under local anesthesia seems to be effective, safe showing good long-term results and feasable in the big majority of patients in a day surgery setting. Laparoscopic approaches should be considered, in this special cohort of patients, as the very second choice.
